# Using an Invasive Plant (Japanese Knotweed) for Mycelium-Based Thermal Insulation Composites

**DOI:** 10.3390/ma19030468

**Published:** 2026-01-24

**Authors:** Kobe Deckx, Joris Verhelst, François Rineau

**Affiliations:** Environmental Biology, Centre for Environmental Sciences, Hasselt University, 3590 Diepenbeek, Belgium

**Keywords:** mycomaterial, invasive plant, *Reynoutria japonica*, thermal insulation, mycocomposites, density, water absorption

## Abstract

**Highlights:**

**What are the main findings?**

- Mycomaterials were successfully made using *Reynoutria japonica* as a substrate.
- Their thermal insulation potential matched ones made from hemp or even PIR.
- However, they had higher density and double the water absorption compared to hemp.

**What are the implications of the main findings?**

- Using invasive biomass reduces land-use competition and regulatory issues linked to hemp.
- Using *R. japonica* turns an ecological threat into a value-added sustainable resource.
- High hydrophilicity suggests that *R. japonica* MBCs are best for moisture-controlled environments.

**Abstract:**

Mycelium-based composites (MBCs)—biomaterials made from fungal-inoculated substrates—are promising candidates to replace conventional rigid thermal insulation panels. However, many MBCs are made from hemp, a plant material that is quite difficult to source in many countries for regulation reasons, and mobilizes agricultural fields at the expense of food and feed crops. Meanwhile, many of our natural and urban ecosystems are subject to invasion by plants that are just burnt or even left in place, while they may be very good substrate for MBCs. This study investigated the comparative physical and thermal properties of MBCs derived from two distinct lignocellulosic feedstocks: hemp shives (a traditional material) and biomass from the highly invasive species *Reynoutria japonica*. Polyisocyanurate (PIR) was included as a synthetic benchmark. The MBCs produced from *R. japonica* demonstrated as low a thermal conductivity as the hemp MBCs in our internally developed method, but also as the PIR standard. However, they exhibited suboptimal physical characteristics: higher bulk density (166 vs. 128 kg/m^3^ for hemp) and significantly higher water absorption (7.5% vs. 3.5% volume uptake after 2 min). This suggest that they are a less viable alternative to hemp-based MBCs for heat insulation applications.

## 1. Introduction

Climate change mitigation requires a significant reduction in greenhouse gas (GHG) emissions, with the energy sector—particularly household heating—being a major contributor. As a result, improving heat insulation in buildings is seen as a highly effective strategy for reducing household energy consumption. However, the materials currently used for insulation are often unsustainable. Polyurethane (PUR) and extruded polystyrene (XPS) are derived from petroleum, making them non-biodegradable, while mineral wools, such as fiberglass and rock wool, are highly energy-intensive to produce and are also non-biodegradable [1]. Some of these materials can even pose health risks, such as skin irritation from fiberglass.

To address these concerns, a transition to more sustainable insulation materials is necessary. Existing alternatives like hemp, cotton, and wool offer biodegradable and renewable options [2] but come with challenges such as limited availability, competition for arable land (especially in the case of cotton and hemp), and high costs. Particularly promising alternatives are mycomaterials—insulation materials made from fungal mycelium grown on lignocellulosic substrates [3]. These materials demonstrate good thermal insulation properties, natural hydrophobicity [4,5], biodegradability (as fungal mycelium usually degrades in a matter of weeks) [6], low flammability [1], low density [7], and excellent soundproofing [8], making them a compelling solution for sustainable insulation. Many studies have shown that the heat conductance values of mycocomposites are in the same range as traditional insulation materials (0.029–0.070 W/m K) [9,10,11,12,13,14,15]. Beyond their potential to valorize waste, MBCs can exhibit thermal conductivity values comparable to conventional synthetic insulation materials such as XPS.

A critical barrier to the large-scale production of MBCs is the sustainability and availability of their feedstocks. Most current substrates either possess low macro-scale availability or are sourced from agricultural lands, leading to direct competition with food and feed production. For instance, while sawdust is a common waste product of wood processing, its widespread use for MBCs creates competition with established, high-demand sectors such as the heating pellet industry [16]. Similarly, substrates like cotton waste are already recycled as industrial absorbents, concrete additives, or plastic fillers. Furthermore, although hemp is recognized as a highly suitable substrate [11,17], its cultivation remains limited globally by regulatory restrictions and is rarely grown on marginal (non-agricultural) land. Consequently, achieving large-scale MBC production necessitates the identification of alternative feedstocks to alleviate the intense competition currently inherent in existing substrate re-use streams.

Conversely, a substantial and non-competitive supply of lignocellulosic material is readily available in many natural and urban environments, derived from invasive plant species. This biomass is often disposed of through simple burning or left in situ to decompose. Repurposing this ecologically disruptive resource for MBC production effectively eliminates competition with established re-use streams. *Reynoutria japonica* (Japanese knotweed), one of the world’s 100 most significant invasive species [18], exemplifies this opportunity. Its aggressive spread, particularly along disturbed habitats like roadways, yields abundant, highly lignified biomass [18,19], and its potential applications are increasingly being explored, ranging from bioremediation (e.g., the binding of dyes and pollutants: [20]) to its use as a bioenergy feedstock [21], paper coatings [22], or a source of high-value biomolecules [23,24]. This lignocellulosic material is highly amenable to fungal degradation, establishing it as an excellent feedstock for mycomaterials. However, its rich tannins and polyphenol content might reduce the fungal colonization rate [19], which may increase the density and reduce the porosity (and consequently thermal performance) of the resulting MBC.

In the present study, we evaluated and compared the key physical properties (thermal conductivity, density, and water absorption) of rigid MBC materials synthesized using a novel, non-competitive feedstock, the invasive species *R. japonica*, and benchmarked against MBCs grown on a conventional substrate, hemp shives. Polyisocyanurate (PIR) foam was included as a commercial insulation material benchmark. The high lignin content typical of the *R. japonica* stem biomass was expected to yield MBCs with a less favorable physicochemical profile for thermal insulation relative to conventional hemp shives. To circumvent this limitation and deliberately target a performance profile similar to the hemp benchmark, the study focused exclusively on the less lignified leaf material of *R. japonica* as the substrate.

## 2. Materials and Methods

### 2.1. Fungal Strain Maintenance

The fungal strain used in this study was the same as that in [15]. It was collected in October 2023 in Spa (GPS 50.4735, 5.8839 Lambert 1972 257542 130281) and belonged to the species *Daedalopsis tricolor*. It was maintained in malt extract agar (MEA: 20 g glucose, 20 g bac/plant agar, 10 g peptone, 20 g malt extract in 1 L distilled water) at 23 °C (Thermo scientific Heraeus incubator, Thermo Fisher Scientific, Waltham, MA, USA) in Petri dishes sealed with parafilm (as the relative humidity in the incubator was not controlled).

### 2.2. Spawn Preparation

Wheat grains (Mill&Mix) were soaked overnight in distilled water in a 2 L measuring cylinder (VWR I213-1129, VWR, Radnor, PA, USA) sealed with aluminum foil (VWR). After draining the water, we autoclaved the grains (HG-80, Hirayama Manufacturing Corp., Kasukabe, Saitama, Japan) for 20 min at 120 °C and inoculated them under sterile conditions with previously grown mycelium with 5 mm × 5 mm mycelium squares from the outer 1 cm of colonies of *D. tricolor* grown on Petri dishes. This spawn was placed in sterile Petri dishes, sealed with parafilm, and incubated at 30 °C for 18 days.

### 2.3. Substrate Preparation

Two substrate types were used for MBC production: *Cannabis sativa* shives and *R. japonica* leaves. Hemp was collected from the field, dried overnight in a stove at 60 °C (estimated residual water content < 5%), and stored dry at ambient temperature before the beginning of the experiment (less than a year). Shives were shredded with a Retsch SM-100 machine using a filter with a mesh size of 4 mm × 4 mm. The shredded substrate was, however, rather heterogenous and made of broad range of particle sizes, from <1 mm to 4 mm. The shredded hemp was soaked in distilled water (50% *v*/*v*) for 1 h, drained, and sterilized by autoclaving.

*R. japonica* biomass was collected on 4 September 2023 near the water treatment station in Halen (50.9583315, 5.1147879) on a river bank where the Grote Gete flows into the Demer. We sorted out the leaf material, then dried it for 24 h at 70 °C (estimated residual water content < 5%), and shredded it, before it was stored dry at ambient temperature before the beginning of the experiment (less than a year). The shredding and sterilization procedure was the same as for the hemp substrate.

### 2.4. Composite Preparation

The spawn and substrate were mixed (20/80% *v*/*v*) in a 2 L measuring cylinder under sterile conditions and placed in a growth container (Unipak 5098-8, Unipak A/S, Galten, Denmark) of 210 mL and a diameter of 95 mm with a SacO2 Type 12 filter (SacO2, Nevele, Belgium) integrated in the lid. We prepared 5 replicates for both hemp and *R. japonica*. After 2.5 weeks of growth, the MBCs were inactivated by placing them at 60 °C for a total of 40 h and 40 min under a plate with a 4 kg weight on top for compression (see Figure S1 in the Supplementary Materials). The resulting materials had a cylindrical shape of 9 cm diameter and 5 cm thickness and were stored in a dry place at room temperature for 94 days. We wanted to benchmark the MBCs with PIR, a good-performing, conventional insulation material. The PIR materials were cut out of an IKO enertherm comfort insulation panel (IKO enertherm Comfort, IKO Group, Antwerp, Belgium) by hand (the aluminum layer was removed so that the material would consist of pure PIR) in the same format. For each material type (MBC made on hemp substrate, MBC made on *R. japonica* substrate, PIR), 5 individual samples were produced.

### 2.5. Thickness Measurements

Material thickness was determined against a reference plane using a geodetic tape measure affixed to a rigid support (a straight wooden jig, as illustrated in Figure S2 in the Supplementary Materials). Due to the inherent surface irregularities of the composites, a total of five replicate measurements were recorded for each sample: the maximum and minimum thickness, supplemented by three random point measurements. The arithmetic mean of these five readings, called L, was used to calculate the heat conductivity index.

### 2.6. Thermal Conductivity Measurements

We measured heat conductivity using our internally developed method [15] with slight modifications. The thermal performance was assessed using a transient heat transfer method. This principle involves monitoring the temperature rise within an insulated containment box when its outer surface is exposed to a constant external temperature of 65 °C for a duration of 30 min. The tested material serves as the primary thermal barrier between the temperature sensor inside the box (WA59 Brifit hygrometer, SensorBlue, Shanghai, China; accuracy **±** 0.5 °C, range from 0 to 50 °C) and the hot environment. A slower internal temperature increase is directly correlated with a lower thermal conductivity (higher heat resistivity) and superior insulating performance. To ensure the tested material was the sole determinant of heat transfer, a specialized lid was designed to integrate the MBC samples in an airtight manner, as their surface was often irregular (refer to Figure S3 in the Supplementary Materials for integration details).

To mitigate the impact of slight manufacturing variability inherent to the testing apparatus (boxes and lids), the setup was divided into two independent measurement units, each comprising a box, a lid, and a calibrated temperature sensor. Measurements of different material types were systematically interspersed between these two units, ensuring that the measurement unit itself did not exert a confounding influence on the derived thermal conductivity values for any single material type. Prior to testing, both units underwent internal validation by running the experiment without an integrated sample. The resulting temperature differences over the 30-minute period demonstrated parity, showing no discernible difference beyond two decimal places, confirming the equivalent performance of the two measurement units.

All measurement runs involved the simultaneous placement of both measuring units into an oven maintained at 65 °C for 30.5 min. Real-time temperature dynamics were tracked via the Sensor Blue application to determine the total temperature increase. To guarantee consistent starting conditions, the measuring units (box and sensor only) were allowed to cool for approximately 3 h between runs within a stable room environment (20 °C), ensuring all 8 runs commenced at 20 °C. This recovery period limited the experimental throughput to a maximum of four runs per day. Furthermore, the oven stability across multiple days was verified by running a polyisocyanurate (PIR 3) reference material in the same unit each morning.

To account for variations in sample thickness due to their surface irregularities, we derived a thermal conductivity index (R) for each material. This index was calculated as the product of the measured thickness (L) and the recorded temperature difference (ΔT) across the material according to Equation (1):

(1) R=L×ΔT

A lower R value indicates lower heat conductivity (higher thermal resistance, and therefore better insulation performance). This method offers a valid and robust comparative measure of thermal performance, although it does not yield an absolute thermal conductivity (λ value in W/mK) as defined by the ISO 8302 standard (heat flow meter method) [25].

### 2.7. Density Measurements

The density and water absorption capacity of the MBCs were measured using the protocol of [15] with slight modifications. The bulk density (D) of the samples was determined using the Archimedean water displacement method instead of geometric calculation because of their surface irregularities. The initial dry mass (m1) was recorded using a precision analytical balance (Viper MB 3XS, Mettler Toledo, Greifensee, Switzerland, 0.01 g precision, range 0.01–1500 g). To measure volume, each sample was anchored to the base of a 1 L graduated cylinder (1 L Polypropylene measuring cylinder, VWR International, Radnor, PA, USA) using tape to ensure accurate reading of the position of the water surface. The cylinder was then filled with deionized water to a precise reference volume (Vr = 500 mL). After 2.5 min of submersion, the volume of the displaced water (Vd) was recorded (accuracy 5 mL). To correct for internal porosity and surface absorption, the sample was removed and briefly blotted with absorbing paper. The volume of absorbed water (Va) was calculated from the mass difference of the sample before and after submersion (m2–m1), assuming a water density of 1 g/mL. Furthermore, the volume of water retained by the absorbing paper (Vw) was quantified by weighing the paper before and after use. The corrected sample volume (Vs) was calculated as Equation (2):

(2) Vs=(Vr−Vd)+Va+Vw

The final material density was expressed as Equation (3):

(3) $$D=\frac{m1}{Vs}$$

The density of *R. japonica* substrate was measured by weighing a known volume of the ground dry leaf litter. The density of *C. sativa* was conducted out of a literature study because not enough hemp shives were in stock for this measurement.

### 2.8. Water Absorption Measurements

Relative water absorption (A) was calculated using the absorbed water volume (Va) obtained during the density measurements, normalized by the material volume (Vs), according to Equation (4):

(4) $$A=\frac{Va}{Vs}$$

This index measures the material’s water uptake capacity. A complementary experiment was conducted on the raw substrates to determine their intrinsic water absorption, enabling an assessment of the mycelium’s impact on reducing moisture uptake. The substrate protocol involved weighing the dry substrate (20 mL in a 100 mL cylinder); adding water to the 70 mL mark with a 1-min pause for initial absorption; and finally, rapidly removing excess water after 2.5 min via sieving. The difference between the wet and dry mass provided the absorbed water mass (Va) from which the Relative Water Absorption was calculated for both the substrates and composites.

### 2.9. Statistics

We used the same pipeline for the three response variables (thermal conductivity, density and water absorption): testing for normality or the response variable using the Shapiro–Wilks test (function “shapiro.test()”), testing the effect of substrate type on the response variable using an ANOVA (function: “aov()”) or Kruskal–Wallis rank sum test (function “kruskal.test()”) depending on the outcome of the normality test, and running post hoc pairwise comparisons using the Tukey test (function: “TukeyHSD()”). Statistical analysis was carried out using R 4.2.2 [26], and all functions were from the R “stats” package.

### 2.10. GenAI

Artificial intelligence was used to improve the grammar, spelling, and overall readability of the text.

## 3. Results

### 3.1. Thermal Conductivity

**Figure 1:**
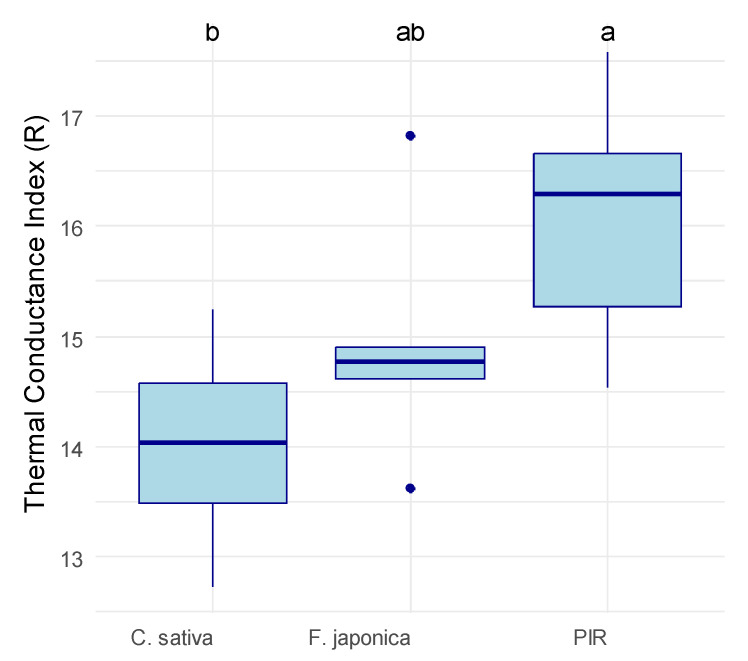
Thermal conductivity index (R) of the tested material samples. The R index was calculated as the product between thickness (cm) and temperature difference (°C): the lower the R, the higher the heat conductance, and the better the material performs for insulation. *C. sativa* = mycelium-based composite with Cannabis sativa shives as an inoculation substrate, *R. japonica* = mycelium-based composite with *R. japonica* leaf litter as an inoculation substrate, PIR = pure polyisocyanurate. The material had a significant impact on the thermal conductivity index according to an ANOVA (*p* = 0.046). Letters on top of the box plots indicate significantly separate groups based on Tukey’s post-hoc test.

The thermal conductivity was significantly affected by the type of material (ANOVA, *p* = 0.046). The MBCs had a slightly lower thermal conductivity index than PIR, with average R index *±* standard deviation of 0.46 *±* 0.04 (*R. japonica*), 0.45 *±* 0.02 (*C. sativa*), and 0.53 *±* 0.05 (PIR) (Figure 1). There was no significant difference between the two MBCs, and both had a significantly lower R index than PIR (Figure 1).

### 3.2. Density

**Figure 2:**
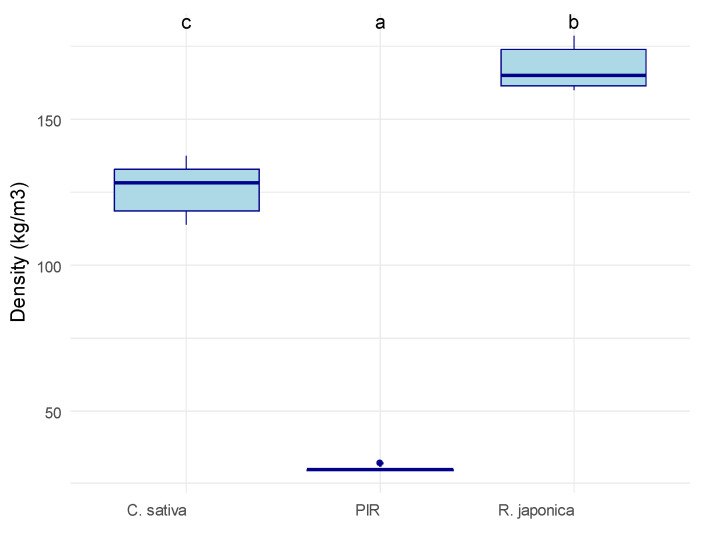
Density of the tested material samples. *C. sativa* = mycelium-based composite with Cannabis sativa shives as an inoculation substrate, *R. japonica* = mycelium-based composite with *R. japonica* leaf litter as an inoculation substrate, PIR = pure polyisocyanurate. The material had a significant impact on density according to a Kruskal–Wallis rank sum test (*p* = 0.001). Letters on top of the box plots indicate significantly separate groups based on Tukey’s post hoc test.

The three categories of the tested materials exhibited low but significantly different densities (Kruskal–Wallis rank sum test, *p* = 0.001), with an average index *±* standard deviation of 166 *±* 7 kg/m^3^ (*R. japonica*), 128 *±* 10 kg/m^3^ (*C. sativa*), and 35 *±* 5 kg/m^3^ (PIR) (Figure 2). The density of the *R. japonica* substrate was as follows: dry = 200 kg/m^3^, wet = 700 kg/m^3^ (after 1 h soaking in distilled water).

### 3.3. Water Absorption

**Figure 3:**
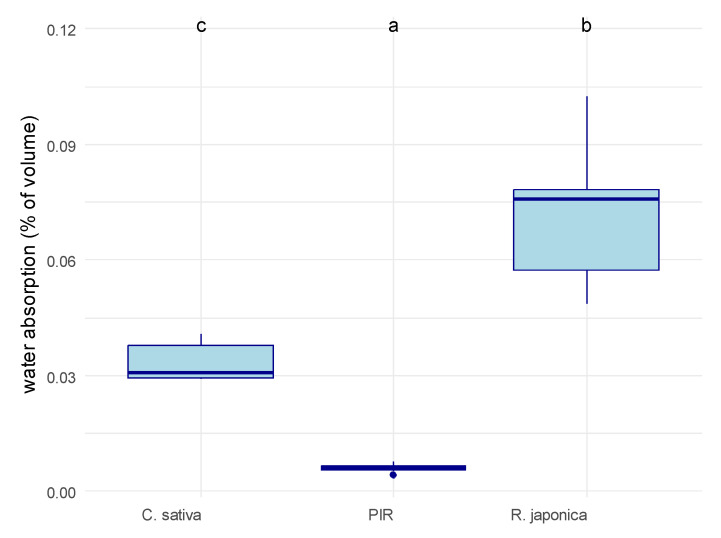
Water absorption of the tested material samples. Water absorption was calculated as %volume of material occupied by water after being submerged for 2 minutes and 30 seconds in water. *C. sativa* = mycelium-based composite with Cannabis sativa shives as an inoculation substrate, *R. japonica* = mycelium-based composite with *R. japonica* leaf litter as an inoculation substrate, PIR = pure polyisocyanurate. The material had a significant impact on water absorption according to an ANOVA (*p* = 8.4 *×* 10^−6^). Letters on top of the box plots indicate significantly separate groups based on Tukey’s post hoc test.

We first measured the water absorption of the substrate alone (Figure 3). The *R. japonica* dried leaves absorbed more water than the *C. sativa* shives (69%: 13.82 mL water/20 mL substrate, 56%: 11.22 mL water/20 mL substrate, respectively). The water absorption was significantly affected by the material type (ANOVA, *p* = 8.4 × 10^−6^). The MBCs made using *R. japonica* as the substrate absorbed significantly more water (7.2 to 2.1%) than the ones made using *C. sativa* (3.3 *±* 0.5%), and the latter, in turn, much more than the PIR materials (0.6 *±* 0.1%).

## 4. Discussion

### 4.1. Good Thermal Performance of the R. japonica MBCs

While the approach used to measure thermal conductivity does not provide the absolute λ values required for certified material datasheets, it serves as a robust tool for relative performance benchmarking. By testing our samples alongside a commercial PIR control under identical laboratory conditions, we can qualitatively assess the competitive standing of these biomaterials. Therefore, the following results should be interpreted as a comparative index of thermal resistance rather than as a quantitative determination of standardized λ coefficients.

Our findings demonstrate that *R. japonica* leaf biomass is an entirely compatible and effective substrate for generating MBCs suitable for thermal insulation applications. This substrate required no adaptation to the standardized bioprocessing procedure established for the widely used hemp shives [27], confirming that the plant does not secrete significant fungicidal or fungistatic compounds that would inhibit fungal colonization.

In terms of thermal performance, MBCs fabricated from *R. japonica* leaves exhibited thermal conductance levels statistically similar to those of the hemp shive MBCs and of PIR (the MBCs made of hemp, however, performed even better than the synthetic material). This is remarkably better than what is reported in the literature on mycomaterials, which typically range between 0.029 and 0.081 W/(m·K) [15,28], hence significantly higher than PIR (0.023–0.028 W/(m·K)). They are, however, consistent with previous work in our lab, and critically, were obtained using the same, unique fungal strain not employed in other research (*Daedalopsis tricolor*) [15]. Our findings clearly show that the fungal strain is a strong determinant of the MBC’s thermal conductivity. Furthermore, they suggest that this specific strain exhibits previously unobserved heat insulation potential. This may be attributable to the specific morphology of its mycelial network. This strain develops a particularly dense mycelium with highly coalesced hyphae, which minimize internal convection. Moreover, it forms a thick, continuous mycelial skin, which potentially restricts air movement inside of the material. Finally, the “fluffy” surface texture likely enhances thermal resistance by creating a stagnant boundary layer of air at the material interface, further reducing convective heat exchange. Collectively, these results strongly support the use of *R. japonica* leaves as a viable, high-performance alternative substrate to hemp shives. Its successful integration into the standard bioprocess confirms that this plant can be utilized as a substrate without compromising the final product’s thermal conductivity.

### 4.2. Higher Density

Despite comparable thermal performance, the MBCs exhibited several suboptimal physical parameters, particularly concerning density. The bulk density of the materials was successfully validated against the established literature values: PIR density (28–32 kg/m^3^) aligned with reported values (30 kg/m^3^), confirming the reliability of our measurement protocol. As anticipated, PIR was significantly lighter, with a density 4- to 6-fold lower than the MBCs. The observed density range for the two MBCs (*C. sativa*: 114–133 kg/m^3^; *R. japonica*:160–179 kg/m^3^) was consistent with other published MBC data [15,29]. However, the *R. japonica* MBCs were approximately 30% denser than the *C. sativa* ones, highlighting the critical influence of the raw substrate on the final composite density. This 30% difference mostly originated from the source material: the *R. japonica* leaf biomass (200 kg/m^3^) was 71% denser than the *C. sativa* hemp shives (117.5 kg/m^3^, [30]). This difference likely comes from the difference in tissue structure between the two materials. Hemp shives are by-products of hemp processing, made almost entirely of fibers of lignified xylem, and hence with large void volume, while the leaf-based bio-mass of *R. japonica* consists of thinner-walled, denser parenchymatous cells, leading to a larger mass-to-volume ratio. Moreover, the morphology of the shredded *R. japonica* leaf biomass is characterized by planar geometries. These two-dimensional particles facilitate a higher packing fraction than elongated fibers, leading to a more consolidated and dense composite matrix. The elevated density of the *R. japonica* MBCs presents potential limitations for practical construction application. First, it increases the load imposed on building supporting structures, potentially requiring costly adaptation, particularly in roof or ceiling applications. Second, it complicates transportation and installation, leading to higher overall project costs.

### 4.3. Higher Water Absorption

Water absorption capacity differed significantly among all material types, successfully confirming the hypothesis that substrate choice dictates material hydrophobicity. The synthetic standard, PIR, exhibited exceptionally low water absorption, retaining only 0.4% to 0.8% of its own volume in water. This was approximately 5- to 10-fold lower than the absorption measured for both the *C. sativa* and *R. japonica* MBCs. Despite the MBCs showing higher overall water uptake than PIR, a crucial finding is the significant enhancement of hydrophobic properties achieved through fungal colonization. The bioprocessing dramatically reduced the hydrophilic nature of the raw substrates: relative water absorption in the raw *C. sativa* substrate dropped from 56% to 2.9–4.1% after colonization. In the raw *R. japonica* substrate, absorption decreased from 69% to 4.9–10%. This considerable reduction is likely attributable to the extensive formation of hydrophobic mycelial biomass, in particular, the formation of a fungal skin on the MBC surfaces [31], which physically repels water. The hierarchy of water absorption is governed by the fundamental chemical composition of the materials: PIR is inherently highly hydrophobic due to its composition of synthetic polymeric monomers. MBC substrates are composed of naturally hydrophilic plant biomass, stemming from their high content of polar biopolymers, primarily cellulose and hemicellulose. The *C. sativa* shives contain a relatively high content of lignin [32]. As a significantly more hydrophobic class of organic polymer compared to (hemi)cellulose, lignin provides higher inherent resistance to water. Conversely, the dried *R. japonica* leaf substrate is expected to possess lower lignin content and higher concentrations of hydrophilic biopolymers, probably explaining its higher water absorption.

This has consequences for real-world applications, where insulation materials are frequently subjected to heat/cold cycles that induce condensation and subsequent water uptake. This moisture ingress not only reduces thermal resistance but also elevates the risk of biological degradation (molding). Consequently, minimizing water absorption is crucial for insulation durability. While the brief water submersion test does not precisely replicate the long-term, dynamic conditions of condensation, these results are a good indicator of performance under extreme exposure. The data confirm that PIR significantly outperformed the MBCs in water resistance, and within the biocomposite group, the *R. japonica*-based MBCs performed significantly worse than their hemp alternative. However, a more construction-relevant interpretation would require a longer-duration or more realistic hygrothermal exposure, such as capillary uptake, over several days. Future studies should therefore focus on assessing sorption behavior and cyclic condensation to better predict material durability in situ.

Collectively, these data confirm that producing MBCs from *R. japonica* biomass is as easy as with any other lignocellulosic substrate and yields materials with the same heat insulation performance, however, their high density and water absorption makes them a less optimal alternative.

### 4.4. Safety and Availability of R. japonica Substrate

We would moreover advise against the widespread use of this species as a commercial substrate due to significant ecological and potential health risks. This plant is globally recognized as a highly invasive species, notorious for causing substantial damage to native biodiversity and infrastructure [33]. Its primary mode of proliferation is highly efficient vegetative spread via rhizome and plant fragments. Consequently, any large-scale harvesting or collection efforts for MBC production carry a critical risk of inadvertently promoting the dispersal of invasive fragments, thereby exacerbating biodiversity loss. Furthermore, efforts to commercially valorize the biomass as an insulation material could unintentionally diminish control efforts aimed at managing the invasive species or create economic incentives for its deliberate cultivation and dissemination. Its use as an MBC substrate therefore represents a significant ecological threat rather than a sustainable service to biodiversity.

While the invasive status of *R. japonica* implies high biomass availability, its practical extraction is complicated by spatial and logistical constraints. Unlike standardized agricultural crops, this species is often found in fragmented patches along riparian zones and roadsides, limiting the use of conventional mechanized harvesting equipment. Although biomass could be sourced from municipal roadside maintenance, the resulting feedstock would likely suffer from high heterogeneity due to mixing with many other plant species. However, diverting this biomass from current disposal methods—such as open-air burning—toward material production offers a ‘waste-to-resource’ opportunity, provided that the increased labor and mobilization costs are offset by the ecological benefits of invasive species management.

## 5. Conclusions

From a practical standpoint, the comparable thermal conductivity index of *R. japonica*-based MBCs to commercial PIR in our in-house test suggests their viability as sustainable alternatives for building insulation. However, their high density and hygroscopicity suggest that their application may be limited to niches such as interior dry-wall partitions or ceiling acoustic panels rather than external cladding or high-humidity environments, where their durability would be compromised by water ingress. Furthermore, while using an invasive species provides an abundant, low-cost substrate, the supply chain must incorporate safe harvesting protocols to eliminate the risk of accidental propagule dispersal. Ultimately, *R. japonica* composites offer a ‘high-performance, high-mass’ alternative that could improve the thermal inertia of lightweight timber constructions, provided that they are structurally strong and protected from moisture.

## Data Availability

The original contributions presented in the study are included in the article/Supplementary Material, further inquiries can be directed to the corresponding author.

## Supplementary Materials

The following supporting information can be downloaded at: https://www.mdpi.com/article/10.3390/ma19030468/s1, Figure S1: Overview of compressive drying method and the diversity of measured big MBCs. The compressive drying occurred in an oven at 60 °C; Figure S2: Height measurement method. A geodesic was taped against a straight and stable wooden construction and the materials were placed against the underside of the geodesic with their flat surface on the table for measurements. Maximum and minimum thickness was measured, in addition to 3 randomly chosen points. The mean of these five values was calculated. This approach was used because of the very uneven surface of most materials; Figure S3: Lids were adapted by gluing silicone gaskets to the inside walls of the lid. Materials were placed in the way that they were throughout their whole perimeter being compressed by the silicone gaskets. The remaining crevices between the two parts of the lid and between the lid and the box were sealed with aluminum insulation tape.

## Abbreviations

The following abbreviations are used in this manuscript:

MBC	Mycelium-based composite
