# Supplementary material for: Using an Invasive Plant (Japanese Knotweed) for Mycelium-Based Thermal Insulation Composites
Source: Materials (Basel). 2026 Jan 24;19(3):468. doi: 10.3390/ma19030468 (PMC12898320; doi:10.3390/ma19030468)
Supplement: Supplementary file 1 [file materials-19-00468-s001.zip › materials-4078081-supplementary.pdf]

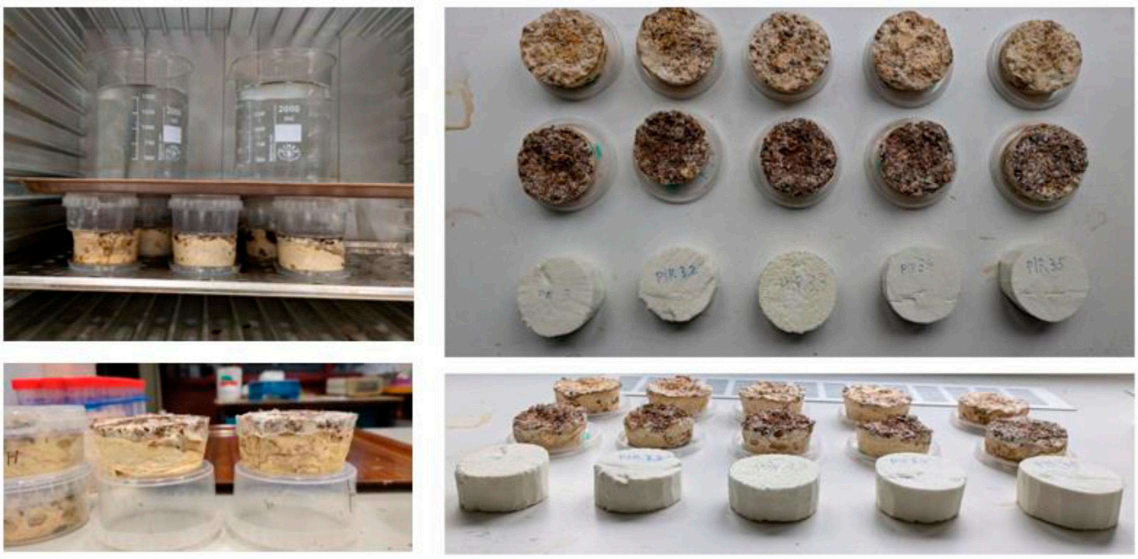

Figure S1. Overview of compressive drying method and the diversity of measured big MBCs. The compressive drying occurred in an oven at 60°C. Top left: MBCs were compressed in between the lids of the growth containers and the containers themselves by placing a plate carrying two measuring cylinders filled with water on top of the growth containers. Normally, one measuring cylinder is used, the second one was used by mistake. As seen in left and right bottom, the materials were therefore a bit over-compressed and did not have a straight cylindrical shape. Left bottom: MBCs coming directly out of the oven, with extra, not dense enough, layer on top resulting from an attempt to equalize the materials' top surfaces. This layer was removed afterward. One can see that the materials were not straight cylinders as a result of the over compression. Top and bottom right: An overview of the measured materials. The picture was made right after the water absorption test. Upper, middle, and lowest rows are respectively the C. sativa MBCs , R. japonica MBCs, and PIR materials. The MBCs had a very uneven surface and the top surface was covered with substrate sticking to the MBC resulting from the failed surface equalization attempt. This layer of substrate was not removed in order to prevent fungal skin damage.

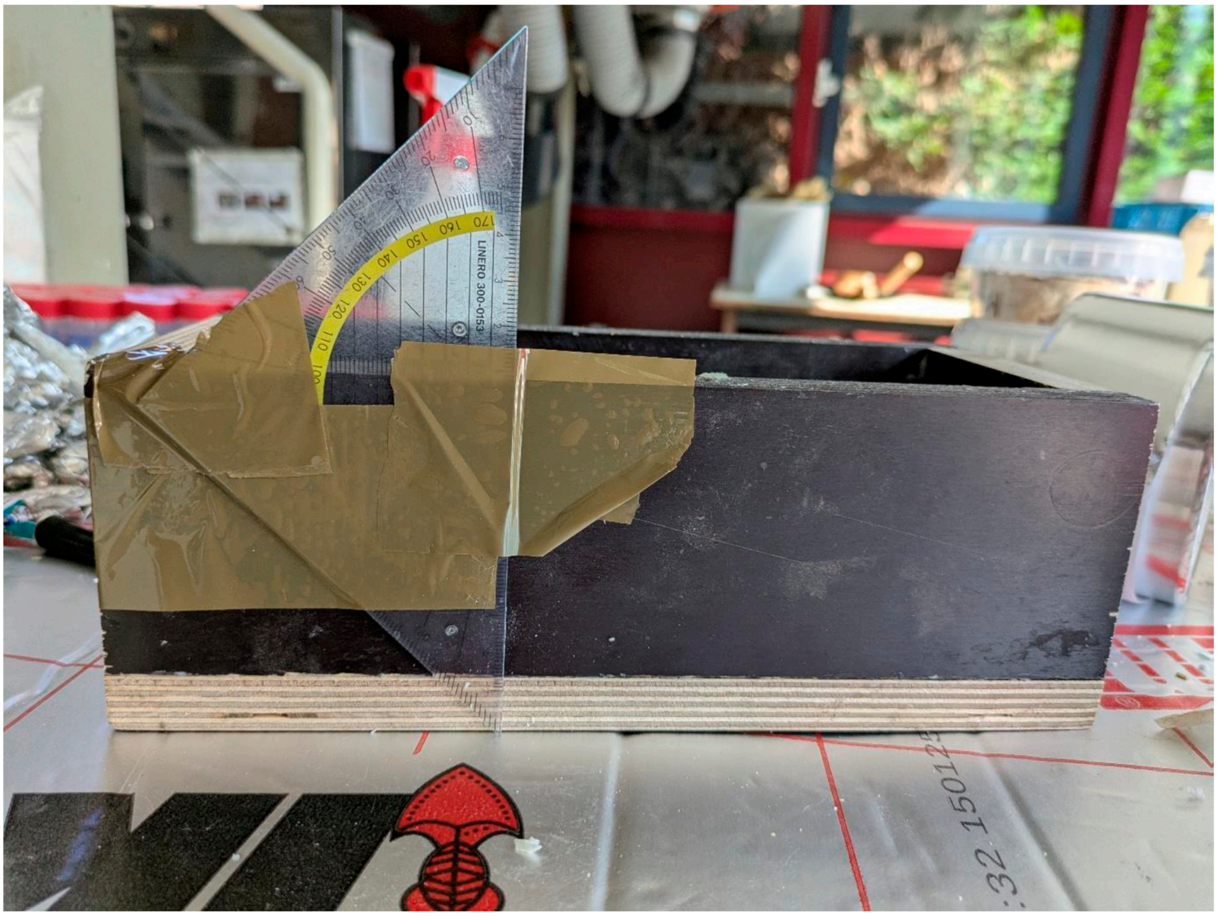

Figure S2. Height measurement method. A geodesic was taped against a straight and stable wooden construction and the materials were placed against the underside of the geodesic with their flat surface on the table for measurements. Maximum and minimum thickness was measured, in addition to 3 randomly chosen points. The mean of these five values was calculated. This approach was used because of the very uneven surface of most materials.

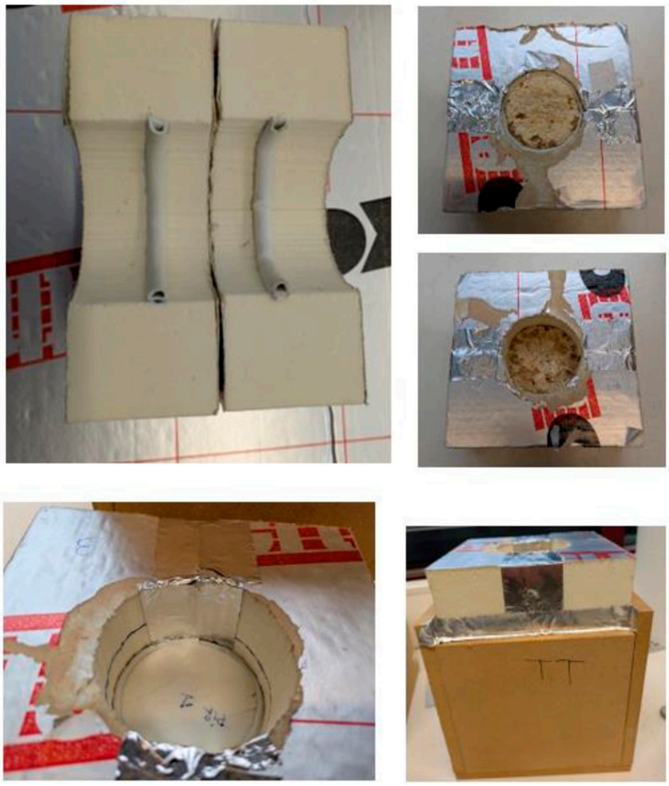

Figure S3. Lids were adapted by gluing silicone gaskets to the inside walls of the lid. Materials were placed in the way that they were throughout their whole perimeter being compressed by the silicone gaskets. The remaining crevices between the two parts of the lid and between the lid and the box were sealed with aluminum insulation tape.
